# Evaluation of Clustering Algorithms on GPU-Based Edge Computing Platforms

**DOI:** 10.3390/s20216335

**Published:** 2020-11-06

**Authors:** José M. Cecilia, Juan-Carlos Cano, Juan Morales-García, Antonio Llanes, Baldomero Imbernón

**Affiliations:** 1 Computer Engineering Department (DISCA), Universitat Politécnica de Valencia (UPV), 46022 Valencia, Spain; jucano@disca.upv.es; 2 Computer Science Department, Universidad Católica de Murcia (UCAM), 30107 Murcia, Spain; jmorales8@alu.ucam.edu (J.M.-G.); allanes@ucam.edu (A.L.); bimbernon@ucam.edu (B.I.)

**Keywords:** clustering algorithms, IoT applications, intelligent systems, edge computing, cloud computing, GPU computing, low-power

## Abstract

Internet of Things (IoT) is becoming a new socioeconomic revolution in which data and immediacy are the main ingredients. IoT generates large datasets on a daily basis but it is currently considered as “dark data”, i.e., data generated but never analyzed. The efficient analysis of this data is mandatory to create intelligent applications for the next generation of IoT applications that benefits society. Artificial Intelligence (AI) techniques are very well suited to identifying hidden patterns and correlations in this data deluge. In particular, clustering algorithms are of the utmost importance for performing exploratory data analysis to identify a set (a.k.a., cluster) of similar objects. Clustering algorithms are computationally heavy workloads and require to be executed on high-performance computing clusters, especially to deal with large datasets. This execution on HPC infrastructures is an energy hungry procedure with additional issues, such as high-latency communications or privacy. Edge computing is a paradigm to enable light-weight computations at the edge of the network that has been proposed recently to solve these issues. In this paper, we provide an in-depth analysis of emergent edge computing architectures that include low-power Graphics Processing Units (GPUs) to speed-up these workloads. Our analysis includes performance and power consumption figures of the latest Nvidia’s AGX Xavier to compare the energy-performance ratio of these low-cost platforms with a high-performance cloud-based counterpart version. Three different clustering algorithms (i.e., k-means, Fuzzy Minimals (FM), and Fuzzy C-Means (FCM)) are designed to be optimally executed on edge and cloud platforms, showing a speed-up factor of up to 11× for the GPU code compared to sequential counterpart versions in the edge platforms and energy savings of up to 150% between the edge computing and HPC platforms.

## 1. Introduction

Societies are advancing guided by the processes of digitalization [1]. These processes are revolutionizing several traditional economic sectors, such as agriculture [2], manufacturing [3], tourism [4], health [5], or even our daily life in the cities [6]. The digital revolution is mainly sustained by two main technological trends: Internet of Things (IoT) and Artificial Intelligence (AI) [7]. The integration of both is mandatory to enable the digital transformation that truly generates benefits for society [8]. AI-enabled IoT (AIoT) brings sensors, machines, cloud-edge computing, analytics, and people together to improve productivity and efficiency, which implies revenue growth and operational efficiency [9].

AI techniques, and particularly, Machine Learning (ML) models are computationally intensive tasks that also require a large amount of high-quality data [10]. This large data is needed to be processed, often in real-time, to extract valuable knowledge that requires access to large computer facilities. Fortunately, over the last decade, there has been tremendous growth in computing power, easily accessible through cloud computing platforms. This growth has been driven by the consolidation of heterogeneous computing where traditional CPUs and hardware accelerators, such as Graphics Processing Units (GPUs), are installed in the same computing platform [11]. The scientific community has been forced to reprogram and even rethought their software to take advantage of this new landscape of computation [12] where parallelism and low-power are the main ingredients.

IoT infrastructures are constantly delivering data in form of data streams, which eventually generate large datasets. These datasets carry hidden patterns, correlations and other valuable insights, the extraction of which can provide a new generation of AI-based application [13]. However, sending the information to the cloud has some limitations, such as high energy consumption rates, high-latency web services, low scalability, privacy policy threats, and transient cloud outages [14]. Edge or fog computing [15] is a recent computing trend that can deal with the aforementioned issues of cloud-based approaches, in which light-weight computations are carried out at the edge of the network, i.e., close to (or actually at) the capture location [16].

Edge computing devices are based on ultra-low-power solutions, such as ARM-based CPUs or on-board microcontroller unit (MCU). Some companies, such as Nvidia, are designing these devices with a higher computational horsepower based on heterogeneous processors. For instance, the Nvidia’s Jetson family [17] include a low-power GPU along with ARM-based processor which can run massively parallel and heterogeneous workloads based on CUDA  [18]. In terms of energy-efficient computing, accelerators substantially reduce application execution time, so that the increased power is amortized. In this work, we analyze these emerging computing devices to figure out whether the performance offered by these platforms is high enough to run computationally heavy workloads and, therefore, new AI-based applications can be executed on them. We focus on clustering algorithms which, in general terms, classify a set of individuals into clusters, with the clusters being created based on distance metrics [19]. These algorithms have been widely used in different areas, such as food industry [20], economy [21], or medicine [22]. A preliminary performance evaluation of clustering algorithms on HPC platforms was presented in Reference [23]. This article substantially differs from Reference [23], as the edge computing platforms are evaluated and compared with HPC ones in terms of both; energy and performance. The main contributions of this paper include the following:

1. Parallel versions of three clustering algorithms are discussed and evaluated. Particularly, we design a GPU parallelization of Fuzzy C- means (FCM) algorithm.
2. An in-depth evaluation of an edge computing platform is performed, showing the benefits of introducing GPU accelerators on edge computing devices for executing heavy workloads. Our results show the inclusion of GPUs in the NVIDIA AGX Xavier can provide performance gains of up to 11× speed-up factor.
3. A performance versus energy comparison is performed between the edge computing and the HPC platforms, reporting an energy savings of up to 150% when using the edge computing platform instead of the HPC counterpart version.

The rest of the paper is organized as follows. Section 2 shows the related work before we briefly introduce the clustering algorithms used for benchmarking and its GPU parallelization using CUDA in Section 3. Then, Section 4 shows performance and energy evaluation under different scenarios. Finally, Section 5 ends the paper with some conclusions and directions for future work.

## 2. Related Work

The parallelization of clustering algorithms has been studied in recent years. There are several works that uses map-reduce approximations on distributed memory clusters to enhance the classification of clustering algorithms. For instance, Xiong [24], Hou [25], and Zhao et al. [26] developed map-reduce solutions using Hadoop as platform to improve the k-means performance applied to different contexts. Woodlet et al. [27] showed a hierarchical data structure, named k-tree, to deal with extremely large data sets. Kwedlo and Czochanski [28] introduced a parallelization approach based on triangle inequality by using MPI and OpenMP on homogeneous clusters, a particular implementation of k-means was provided to avoid unnecessary distance calculations. Liu et al. [29] developed a parallel FCM segmentation algorithm based on Apache Spark for agricultural image analytics.

Some works in the literature have discussed parallel versions of k-means in different platforms, such as mutlicore CPUs, GPUs, and FPGAs  [30,31]. For instance, Li et al. [32] pointed out the density of data as one of the most important factors in terms of GPU performance. They designed different implementations for data sets with high and low dimensionality. Cuomo et al. [33] proposed a GPU parallelization of the k-means algorithm using CUDA, trying to deal with the classic problems of space limitations on the device, and host-device-host data transfers. Another implementation of k-means in CUDA is presented in Reference [31], where the authors compare their CUDA implementation with CPU implementations using OpenMP and Basic Linear Algebra Subroutine (BLAS). Moreover, some GPU implementations have been also provided for FCM algorithm. For instance, All-Ayyoub et al. [34] proposed a brFCM algorithm, a faster variant of the FCM algorithm on GPUs, reporting a speed-up factor of up to 22.43×. However, they develop an image segmentation-based implementation of this algorithm and did not report any energy consumption numbers. Ali et al. [35] also provided GPU implementations of the FCM for image segmentation. They actually developed three methods analyzing different bottlenecks. Finally, there are only few works about the FM parallelization on GPUs [36,37].

All works previously described are approximations from a general point of view, including its design, efficiency, implementations, etc. There are other works that offers applications based on clustering algorithms, such as air pollution detection [38], medical images [39,40], or even monitoring and supervising fault tolerance in Wireless Sensor Networks [41]. However, to the best of our knowledge, no work has been yet proposed to evaluate edge computing platforms to performed heavy workloads, such as clustering algorithms.

## 3. Parallel Clustering Algorithms

Clustering algorithms are iterative procedures where a set of individuals (i.e., points in a multidimensional space) are assigned to clusters (or groups) based on the optimization of an objective function. The objective function can include different measures, such as distance (Euclidean, Mahalanobis, etc.), connectivity, and/or intensity. Many clustering techniques have been proposed in the literature as underlying algorithms for AI-based applications. We refer the reader to Reference [42] for insights.

The main characteristics of a clustering algorithm include: (1) *scalability*, i.e., the ability to manage a growing number of individuals in a limited period of time, (2) *adaptability* to identify different clusters, (3) *self-driven*, i.e., it should require no knowledge of the problem domain, (4) *stability* which means that the algorithm is not influenced in the presence of noise or/and outliers, and (5) *data-independency*, i.e., the algorithm should not be affected by the organization of individuals in the dataset [43].

Clustering algorithms can be divided into hard and soft techniques. The former assigns individuals into a cluster, i.e., an individual can only belong to a cluster. The latter, however, groups individuals into different clusters with a certain probability. The well-known k-means algorithm provides a hard-partition scheme. Leading exemplars of soft clustering are fuzzy algorithms, such as the fuzzy c-means (FCM) algorithm [44] and fuzzy minimals (FM) [36,45]. Table 1 summarizes the main features of each clustering algorithm. Soft clustering refers to whether the grouping of a data or individual is exclusive to a cluster or has some degree of membership with respect to other clusters. Regarding of number of clusters, k-means and FCM need an input parameter to establish the number of clusters to be performed. On the contrary, FM does not need prior knowledge of the number of clusters, and it presents the advantage that the clusters do not have to be Compact Well-Separated (CWS). In what follows, these three clustering algorithms are introduced along with the proposed parallelization approach.

### 3.1. K-Means

K-means is a well-known clustering algorithm that is really heavy from a computational point of view. It is an iterative algorithm that seeks to classify data into clusters or groups depending on a distance function. The result is, therefore, a set of groups in which all individuals belonging to the same group are more similar than those in other groups. The index *k* in k-means refers to the number of clusters to be developed and is an input parameter of the algorithm. The algorithm looks for the prototypes or centro_ids (centroids for short), which will act as the representative of each of cluster. Euclidean distance is used to determine which group an individual belongs to. The k-means clustering algorithm works as follows:

1. *Initialization*: A given number of clusters is established (i.e., *k* parameter), and then *k* centroids are established in the data space. In the initial stage, the centroids are chosen randomly.
2. *Assignment*: Each data point is evaluated among all centroids, and it is eventually assigned to the cluster with its nearest centroid.
3. *Update*: The centroids of each cluster are updated, choosing as the new centroid the position of the average data of that cluster.

Steps 2 and 3 are iterated until the centroids are stable, or at least centroids do not move above a threshold distance in each step. Moreover, the Euclidean distance calculation between points and centroids of each cluster can be fully performed in parallel. Regarding the computational complexity, the k-means clustering algorithm, targeting *d* dimensions datasets is a NP-hard problem in general Euclidean space (*d* dimensions), even for two clusters and NP-hard for a general number of clusters *k*. The problem could be solved in O(ndk), being *k* and *d* fixed and *n* the number of entities to be clustered. Therefore, the k-means algorithm is computationally challenging and thus is well-suited for parallelization in multi and many core systems. In this work, we use the GPU version of CUDA found in the NVIDA RAPIDS library, and we refer the reader to Reference [46] for insights.

#### 3.1.1. Fuzzy C-Means Clustering (FCM)

Fuzzy clustering is a way of clustering where each individual can belong to more than one cluster with different probabilities of belonging. One of the best known fuzzy clustering methods is the FCM Algorithm. The FCM is very similar to the k-means algorithm and is based on minimizing a function (Equation (1) in our case) until an optimal fuzzy partition is obtained.

(1) $$J_{m}\left(U,v\right)=\sum_{k=1}^{n}\sum_{i=1}^{c}{\left(u_{ik}\right)}^{m}d_{ik}^{2}.$$

$d_{ik}^{2}$ is the square distance between the elements and centroids of each cluster, and it is calculated as $d_{ik}^{2}=\left|\right|x_{k}-v_{i}{\left|\right|}_{A}^{2}={\left(x_{k}-v_{i}\right)}^{T}A\left(x_{k}-v_{i}\right)$.

Where

- $X=(x_{1},x_{2},\ldots ,x_{n})\in R$ are the data,
- $v_{i}=\left(v_{i1},v_{i2},\ldots ,v_{in}\right)$, is the vector with centroids of each i-cluster,
- ${\left|||\right|}_{A}$ is the induced norm by *A*, and
- *A* is a positive dimensional weight matrix,

where *A* is the identity matrix and $d_{ik}^{2}$ is the square of the Euclidean distance. The weight associated to each square distance, ${\left(u_{ik}\right)}^{m}$, is the mth power of the k-data degree of membership to cluster *i*. When m→1, the optimal partition is closer and closer to an exclusive partition, while, when m→∞, the optimal partition is closer to the matrix with all its values equal to (1/c). The *m* values normally used are values in the range [1...30]. Each selection of a particular m-value marks a specific Fuzzy C-Means algorithm according to Bezdek [44].

A multicore (GNU C/OPENMP) and GPU (NVIDIA CUDA) FCM implementations are introduced in this article. The sequential baselines of FCM can be formalized in the following steps:

1. Initializate c,m,A,y||A||, choose an initial matrix $U^{0}\in M_{jc}$.
2. Calculate centroids with $v_{i}=\frac{\sum_{k=1}^{n}{\left(u_{ik}\right)}^{m}x_{k}}{\sum_{k=1}^{n}{\left(u_{ik}\right)}^{m}};1\leq i\leq c$.
3. Update the fuzzy partition matrix $U=[U_{ik}]$ with $U_{ik}={\left(\sum_{j=1}^{c}{\left(\frac{d_{ik}}{d_{jk}}\right)}^{\frac{2}{m-1}}\right)}^{-1};1\leq k\leq n;1\leq i\leq c$.
4. If the stop criterion is reached then the execution finishes. Otherwise, return to step 2.

The most common stop criteria are: (a) a maximum number of iterations or (b) the variation in the U-matrix is below a certain threshold $\left|\right|U^{k+1}-U^{K}\left|\right|<\varepsilon$.

The FCM algorithm computational complexity is rather similar than the k-means algorithm. The algorithm runs in O(ndk), where *n* the number of entities to be clustered, the *d* dimensions of the data points and *k* the number of clusters. Most of the time is spent in calculating the fuzzy partition matrix *U*, in which its update requires $O(ndk^{2})$ floating point operations as the calculation of the Euclidean distance introduces another nested summation. There are several strategies to reduce this complexity, but this is out of the article’s scope. We refer the reader to Reference [47] for insights.

The FCM parallelization is described in Algorithm 1. As discussed previously, the FCM algorithm can be divided into several steps, but they have to be performed sequentially as the fuzzy−u matrix obtained from an iteration is provided as an input for the next one. Therefore, the parallel design is focused on accelerating each of these steps on the GPU. In the first step (line 1), the fuzzy_u matrix is set in the GPU with random numbers using curand. The random numbers are stored in the states vector. The body of the loop is basically divided into six different steps. First (line 3), two matrices are prepared, one based on $fuzzy_{u}$ named mf and the other vector_u_transfor. The vector_u_transfor matrix is obtained by multiplying mf matrix with identity matrix using cuBlas library. Then, the numerator and the denominator are obtained (line 4 and 5) to calculate the center matrix of step *i*, using the previous matrix calculated. The center of step *i* (line 6) is obtained, and the distance between the datamatrix and the center (line 7) is calculated. Next, the error obtained in this step is tested which is calculated by reducing the mf matrix using the Thrust library. If the obtained error is less than an input parameter, the execution is finished; otherwise, the algorithm will continue to the next iteration. Finally, the fuzzy_u matrix are updated with the data from the distance matrix (line 12) to carry out with the next iteration.
**Algorithm 1** FCM algorithm in GPU.1:init_u<<<bl,th>>>(states,fuzzy_u,clusters,rows);2:**for**step=1;i<MAX_STEPS;i=i+1**do**3: $cublasDgemm\_configuration_{u}\left(fuzzy\_u,mf,vector\_u\_transform,clusters,rows,columns\right);$4: $numerator\_Centroid_{i}<<<bl,th>>>\left(numerator,mf,columns,rows,clusters\right)$5: $determinator\_Centroid_{i}<<<bl,th>>>\left(denominator,vector\_u\_transform,columns,rows,clusters\right)$6: obtain_centers<<<bl,th>>>(center,numerator,denominator,mf,clusters,rows,columns)7: $distance\_matrix<<<bl,th>>>(distance,center,data_{m}atrix,rows,columns)$8: $Error\_step_{i}=thrust::reduce\left(mf\right);$9: **if** $(Error\_step_{i}-Error\_step\_(i-1))<error$ **then**10:  break;11: **end if**12: new_u_for_next_iteration<<<bl,th>>>(fuzzy_u,distance,rows,columns,clusters);13:**end for**14:cudaMemcpy (u_host,fuzzy_u,cluster∗rows∗sizeof(FLOAT), cudaMemcpyDeviceToHost);

Additional considerations of our FCM implementation using CUDA include the following:

1. *Use of Pinned Host Memory*. The host data assignments (CPU) are paginated by default. The GPU cannot access data directly from the host memory, so, when a data transfer from host memory to device memory is called, the CUDA controller must first map a pinned host array, copy the host data to the pinned array, and then transfer the data from the pinned array to the device memory. The pinned memory is used as a storage area for transfers from the device to the host. We can avoid the cost of transferring between paged host memory and pinned memory by directly assigning our host arrays to the pinned memory. In this case, memory reservation is done with *cudaMallocHost*, instead of *malloc* and *calloc*, and to free memory *cudaFreeHost* is used.
2. *Matrix multiplication*. It is performed by calling cublasDgemm, a function in the CUDA Basic Linear Algebra Subroutine library (cuBLAS). This same library is also used for the sum of columns in an array, as the sum of rows or columns in an array can be seen as a matrix-vector multiplication, where the elements of the vector are all ones. Through these implementations, it was possible to drastically reduce the execution time in the calculations of the algorithm, reaching speed-ups that are discussed in Section 4.

#### 3.1.2. Fuzzy Minimals (FM)

The FM algorithm is a fuzzy clustering technique like FCM, but it does not require any input parameter. For an in-depth study of the FM algorithm, we refer the reader to Reference [36,45], and the authors also carried out a detailed study of the algorithm in Reference [37]. We now present an overview of the FM algorithm. Algorithm 2 outlines the FM algorithm where two main procedures are identified, i.e., (1) the calculation of the *r* factor (see Equation (2)) and (2) the calculation of prototypes or centroids (see Algorithm 3). The *r* factor can be described as a parameter to measure the data set isotropy. FM assumes the use of Euclidean distance that implies homogeneity and isotropy of the features space. If such homogeneity and isotropy are broken, then clusters are created in the features space.

(2) $$\frac{\sqrt{|C^{-1}|}}{nr^{F}}\sum_{x\in X}\frac{1}{1+r^{2}d_{xm}^{2}}=1.$$

Equation (2) shows the factor *r* equation that is based on a non-linear expression. $|C^{-1}|$ is the determinant of the inverse of the covariance matrix, *m* is the mean of the sample *X*, $d_{xm}$ is the Euclidean distance between *x* and *m*, and *n* is the number of elements of the sample.
**Algorithm 2** The FM algorithm, where *X* is the input dataset to be classified, *V* is the algorithm output that contains the prototypes found by the clustering process. *F* is the dimension of the vector space.1:Choose $\varepsilon_{1}$ and $\varepsilon_{2}$ standard parameters.2:Initialize $V=\left\{\;\right\}\subset R^{F}$.3:Load_Dataset(X)4:r=Calculate_r_Factor(X)5:$Calculate\_Prototypes(X,r,\varepsilon_{1},\varepsilon_{2},V)$

**Algorithm 3**Calculate_Prototypes() of FM algorithm.
1:
**for** k=1;k<n;k=k+1 **do**
2: $v_{\left(0\right)}=x_{k}$, t=0, $E_{\left(0\right)}=1$3: **while** $E_{\left(t\right)}\geq \varepsilon_{1}$ **do**4:  t=t+15:  $\mu_{xv}=\frac{1}{1+r^{2}\cdot d_{xv}^{2}}$, using $v_{\left(t-1\right)}$6:  $v_{\left(t\right)}=$$\frac{\sum_{x\in X}{\left(\mu_{xv}^{\left(t\right)}\right)}^{2}\cdot x}{{\left(\mu_{xv}^{\left(t\right)}\right)}^{2}}$7:  $E_{\left(t\right)}=\sum_{\alpha =1}^{F}\left(v_{\left(t\right)}^{\alpha }-v_{\left(t-1\right)}^{\alpha }\right)$8: **end while**9: **if** $\sum_{\alpha }^{F}\left(v^{\alpha }-w^{\alpha }\right)>\varepsilon_{2}$, ∀w∈V **then**10:  $V\equiv V+\left\{v\right\}$.11: **end if**12:
**end for**



Next, the algorithm calculates centroids or prototypes. This is an iterative procedure that aims to minimize an objective function shown in Equation (3).

(3) $$J_{\left(v\right)}=\sum_{x\in X}\mu_{xv}\cdot d_{xv}^{2},$$

where

(4) $$\mu_{xv}=\frac{1}{1+r^{2}\cdot d_{xv}^{2}}.$$

Finally, $\varepsilon_{1}$ and $\varepsilon_{2}$ are input parameters which establish the error degree committed in the minimum estimation and show the difference between potential minimums, respectively.

(5) $$v=\frac{\sum_{x\in X}\mu_{xv}^{2}\cdot x}{\sum_{x\in X}\mu_{xv}^{2}}.$$

The FM computational complexity is rather similar than the FCM and k-means algorithms. However, it has two main steps that should be analyzed separately. The factor *r* runs in O(nd) where *n* the number of entities to be clustered and the *d* dimensions of the data points. Indeed, the calculation of the covariance matrix and its determinant are the additional calculation to be performed for each data point. The prototype calculation is again O(nd2), where the execution time is spent in calculating the prototype calculation of each partition *C*. It is important to note that the number of clusters *k* is not required in this algorithm, but it would require to calculate the Error ($E_{\left(t\right)}$) for each data point of dimension *d*.

Algorithms 4 and 5 show the GPU implementation of FM algorithm previously presented in Reference [37]. The parallelization of the factor *r* procedure is based on the parallelization of the calculation of the fuzzy covariance matrix. This is translated into two CUDA kernels. The former is the calculation of the covariance matrix and the latter is the calculation of its determinant. The number of iterations is determined by the number of rows in the data set. However, the performance is also penalized by the number of columns.
**Algorithm 4***R* Factor calculation algorithm in GPU1:**for**i=1;i<rows;i=i+1**do**2: $covariance<<<bl,th,sh>>>(dataset,determ,row_{i},rows,cols)$3: detvalue=cusolver_thrust(determ)4: $rfactor+=\frac{1}{\sqrt{detvalue}}$5:**end for**

**Algorithm 5** Covariance (dataset,determ, p, rows,cols)
1:
**for** i=1;i<cols;i=i+1 **do**
2: **for** j=1;j<cols;j=j+1 **do**3:  sum=04:  **for** k=1;k<rows;k=k+1 **do**5:   sum+=(dataset[k][i]−p[i])*(dataset[k][j]−p[j]);6:  **end for**7:  determ[j][i]=sum/rows;8: **end for**9:
**end for**



## 4. Evaluation and Discussion

This section shows an experimental evaluation of the clustering algorithms presented above (i.e., k-means, FCM, and FM). First, the hardware and software environment in which the experiments are performed are introduced. Furthermore, the datasets used for the experiments are described, highlighting the main configuration parameters that can affect the performance of the clustering algorithms. Finally, this section ends with a performance and energy evaluation on the different targeted architectures, i.e., HPC and edge computing platforms. We analyze the CPU and GPU versions of all the clustering algorithms under study in each platform individually and then evaluate a trade-off on both platforms.

### 4.1. Hardware Environment and Benchmarking

**Figure 1:**
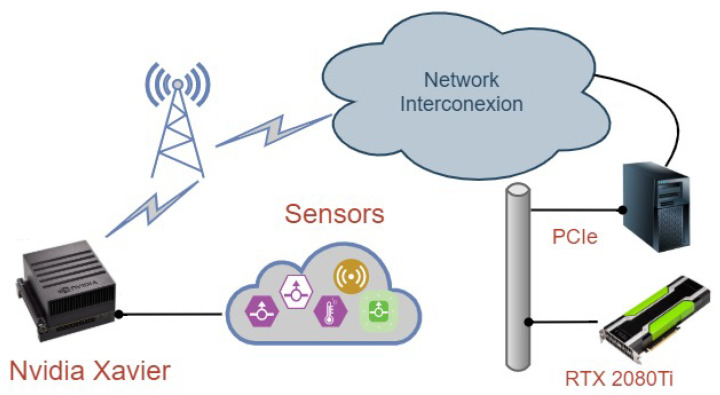
The system infrastructure in a nutshell.

As previously explained, the main objective of this paper is to validate edge computing devices as a compelling alternative for running AI workloads. Therefore, a performance comparison between an HPC infrastructure and the most powerful edge computing device on the market can shed light on the extent to which these platforms can support heavy workloads. Figure 1 shows the network infrastructure. As observed, it consists of several elements, including the sensing devices, the communication concentrator and the cloud. The sensing devices periodically collect data that is sent to the communication concentrator where the edge computing infrastructure would be placed. This communication concentrator can directly send raw information to the cloud for further analysis. In this case, this module would only be equipped with communication technologies, such as LPWAN (LoRaWAN), WiFi, or cellular networks, e.g., 4G/5G. However, if the clustering algorithms are performed at the edge, this communication concentrator would also include an edge computing device, such as the Nvidia Jetson Xavier. In this latter case, the communication concentrator would only send the clustering result to the cloud if necessary.

With this in mind, the particular hardware infrastructure used for our experiments is as follows. The HPC platform that would be placed in the cloud is an Intel-based architecture; composed of an Intel Xeon(R) Silver 4216 CPU processor with sixteen physical cores (thirty-two threads) running at 2.10 GHz with a maximum of 3.20 GHz. It has 32 MB of shared L3 cache. It offers support for SSE 4.2 (128-bit registers), AVX2 (Advanced Vector Extensions) with 256-bit registers and AVX-512 (512-bit registers) with one FMA (Fuse Multiply ADD). This platform also includes a NVIDIA GPU GeForce RTX 2080 Ti (Turing family), with Compute Capability 7.5, 4352 CUDA Cores (68 SM and 64 CUDA Cores per SM), 12 Global Memory DDR5 with 352 Memory Bus, and 48 KB of shared memory per block. The edge computing platform is the NVIDIA Jetson AGX Xavier which has 8-core NVIDIA Carmel ARM v8.2 64-bit CPU, 8MB L2 + 4MB L3, 512-core Volta GPU with 64 Tensor Cores and 32GB 256-Bit LPDDR4x running at 136.5 GB/s. The peak power consumption is between 10 W–30 W according to its specifications (https://developer.nvidia.com/embedded/develop/hardware).

In order to calculate the energy consumption of our system, we measured, at intervals of one second, the power consumed by each of the devices used. The power consumed by the NVIDIA Jetson AGX Xavier was measured using the Watts Up Pro power meter. Regarding to the HPC platform, the power consumption was measured using the NVIDIA Management Library (NVML).

A set of numerical benchmarks are used to evaluate the performance of the three clustering algorithms. These benchmarks are made up of 100 K points with 80 columns each corresponding to five hyper-ellipsoids $S_{k}$, with $S_{k}\subset R^{80}$, ∀k∈{1,2,3,4,5}, and $S_{i}\cap S_{j}\forall i\neq j$. The cardinal of each subset is: $|S_{1}|=20,868$, $|S_{2}|=20,104$, $|S_{3}|=19,874$, $|S_{4}|=22,380$, $|S_{5}|=16,774$. Note that there are different parameters that can affect the clustering algorithm performance. They are columns, rows, and number of clusters. Columns refer to different variables for each element that should be clustered. Rows, however, represent different instances of the elements to be classified. Finally, some clustering algorithms require as an input the number of clusters to be performed; thus, this parameter can also affect performance.

From this dataset, three different experiments are carried out to evaluate the impact of these parameters. The first one (Experiment 1) consists of 100 K rows and 2, 4, 8, 16, 32, 64, and 80 columns, respectively. The columns are progressively increased to evaluate the scalability. The second experiment (Experiment 2) varies the number of rows in the range of $10^{2}$, $10^{3}$, $10^{4}$ and $10^{5}$. The last experiment (Experiment 3) uses all available data (100 K rows and 80 columns) varying the number of clusters (2, 4, 8, 16, 32, 64, 128, 256, 512, and 1024). Finally, the convergence criteria established in the clustering algorithms is the number of iterations for k-means (50 iterations) and FCM (100 iterations) algorithms and a given error for the FM algorithm (e1 = 0.000001). These convergence criteria is exactly the same in all experiments.

### 4.2. Performance Evaluation

This section shows the performance evaluation of the three clustering algorithms on both targeted platforms, i.e., HPC and edge computing platforms. First, each platform is evaluated separately to figure out the performance gap between its CPU and GPU implementations. Then, the HPC and edge computing platforms are compared in terms of performance and energy consumption.

#### 4.2.1. HPC Platform

**Figure 2:**
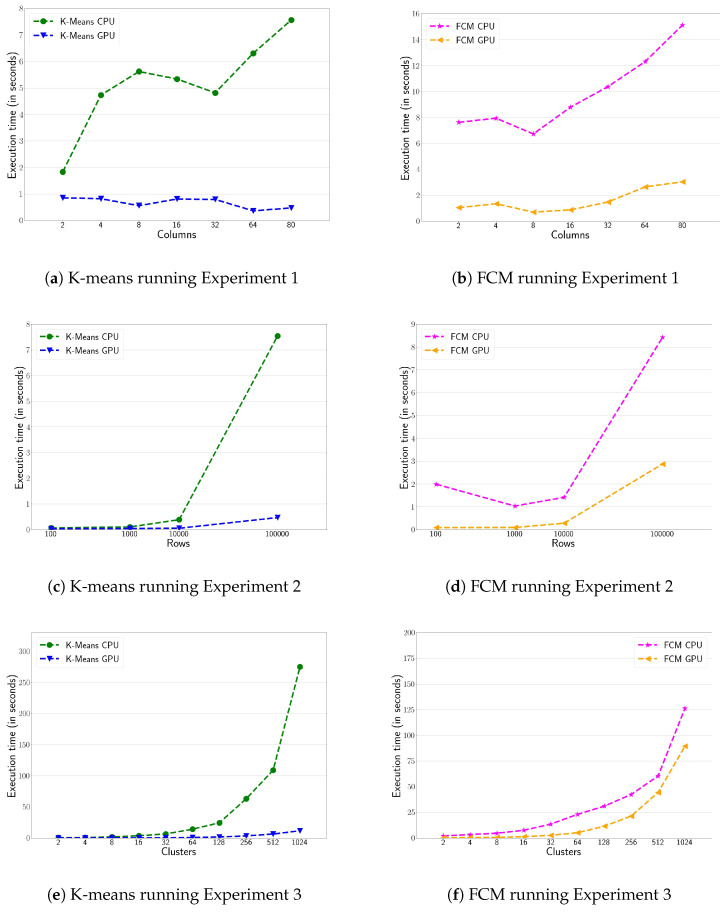
Execution time (in seconds) of the clustering of the k-means (right-hand side) and Fuzzy C-Means (FCM) (left-hand side) algorithms for the three experiments described in Section 4.1. GPU and CPU versions are executed in the HPC platform.

Figure 2 shows the performance evaluation of k-means and FCM clustering algorithms on the HPC platform. The multicore and CUDA versions are executed on the CPU and GPU, respectively. Figure 2a,b show the execution time for the first experiment, i.e., increasing the number of variables (i.e., columns). The massively parallel version of the CUDA implementations, defeat by a wide margin their multicore CPU counterpart versions (i.e., OpenMP). A speed-up factor of up to 17× for k-means and 10× for FCM are reported, also showing that the GPU codes achieve a linear scalability along with the number of variables. Figure 2c,d show the execution time, increasing the number of rows (i.e., Experiment 2). Again, the GPU version defeat CPU counterpart version by a wide margin. Actually, the performance gap increases along with the number of rows. In particular, the GPU version of k-means obtains a performance of up to 16× compared to its CPU implementation (see Figure 2c). Moreover, the GPU version of FCM obtains 11× speed-up factor for values lower than 10 K. Figure 2d shows that as the number of rows increases the performance gap decreases to 3× for 100 K rows. Indeed, the FCM algorithm is more affected by increasing the number of columns than the number of rows. Finally, Figure 2e,f show the impact on performance by varying the number of clusters in both k-means and FCM (i.e., Experiment 3). Both clustering algorithms have an input parameter to determine the number of clusters to be developed. Figure 2e shows 24× speed-up factor of the k-means’ GPU version compared to its sequential counterpart version. However, the performance gap between CPU and GPU is smaller for FCM (see Figure 2f), reaching up to 2× speed-up factor. This is actually the same for the k-means algorithm when the number of clusters is lower than 256. As long as the number of clusters increases, the GPU occupancy also increases since more parallelism (i.e., CUDA thread blocks running in parallel) is available. However, the FCM has a higher float operations intensity since it has to calculate the probability of belonging to each group which reduces the GPU horsepower.

**Figure 3:**
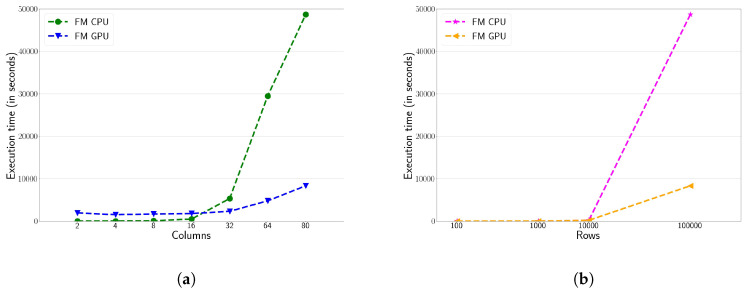
Execution time (in seconds) of Fuzzy Minimals (FM) algorithm for the first (**a**) and second (**b**) experiment on the HPC platform, comparing both CPU and GPU versions.

Finally, the performance evaluation of the FM algorithm on CPU and GPU is shown in Figure 3. Here, it is reported the execution times for the first and second experiments. As previously explained in Section 3, the FM algorithm does not require the number of clusters as an input parameter. As in the previous cases, the GPU implementation offers better performance than the CPU implementation, especially when the workload is large enough. Figure 3a shows the performance of the first experiment. It can be observed, the CPU shows better performance than the GPU when the number of columns or variables is less than 32. When this value increases, the performance of the CPU decreases rapidly to the benefit of the GPU. In the first experiment, the GPU version of FM obtains 6× of speed-up factor compared to its sequential counterpart version when all columns are targeted. Figure 3b shows the performance of the FM algorithm running the Experiment 2. In this case, the number of records to process in this algorithm is critical. Figure 3 shows a significant drop in CPU performance from 10 K rows. Overall, we can conclude that the FM algorithm achieves better performance results with the GPU for higher computational workloads.

#### 4.2.2. Edge Computing Platform

**Figure 4:**
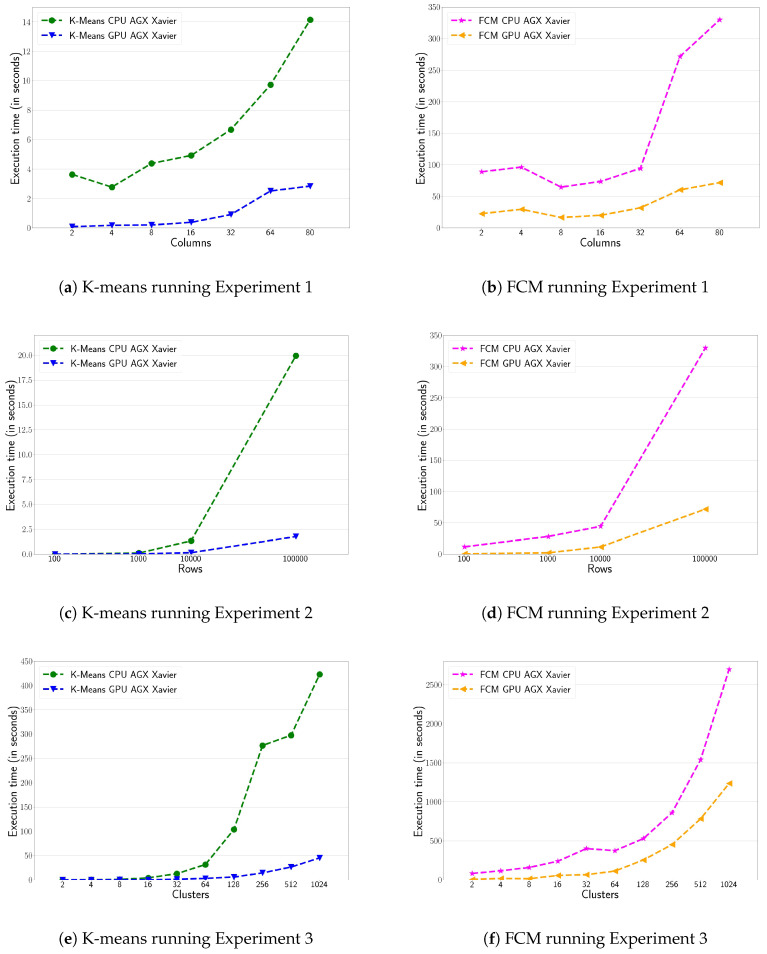
Execution time (in seconds) for the three benchmarks for K-means and FCM algorithms on the NVIDIA AGX Xavier.

This section evaluates the edge computing platform, running the GPU and CPU version of the three clustering algorithms under study. Figure 4 shows the performance evaluation on the NVIDIA AGX Xavier. The general conclusions are quite similar to those obtained in the analysis of the HPC platform. Figure 4a,b show the performance of the k-means and FCM algorithms, running the first experiment. Again, the GPU defeat CPU by a wide margin, reaching up to 5.5× speed-up factor. The speed-up factor reported here for the GPU is lower than in the HPC platform. Indeed, the GPU plugged into the NVIDIA Xavier is a low-power device that only includes a Stream Multiprocessor (SM); this limits the number of CUDA blocks executed in parallel and, thus, the overall CUDA application performance. Figure 4c,d show the performance of the k-means and FCM algorithms, running the second experiment on AGX Xavier. As in the HPC infrastructure, GPU implementations offer better performance than CPU ones. The performance figures reach values close to 10× of speed-up factor when targeting the maximum number of rows simulated. Figure 4d shows the FCM performance differences between Xavier’s CPU and GPU. This difference is higher than the one achieved in the HPC infrastructure, reaching up to 5× of speed-up factor between both implementations. Finally, Figure 4e,f show the performance running the third experiment. As in the case of the HPC platform, the GPU implementation offers better performance when the number of clusters is increased in both algorithms. The k-means can obtain performance differences up 8× speed-up factor when they deal with the maximum number of clusters targeted. The FCM performance in Figure 4f follows the same behaviour as in the HPC platform with a speed-up close to 1.5×.

**Figure 5:**
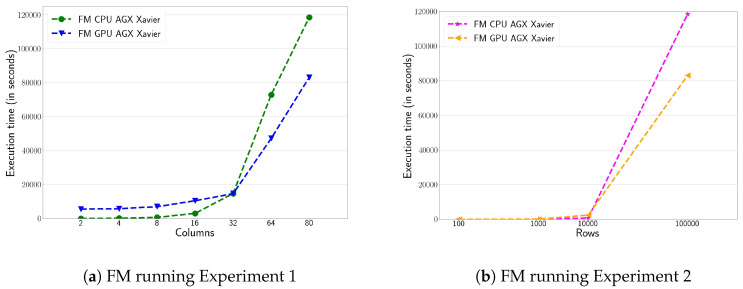
Execution time (in seconds) for Experiment 1 and 2 for the FM algorithm on the NVIDIA AGX Xavier.

Figure 5 shows the FM clustering performance on the AGX Xavier. Once again, the conclusions are quite similar to those obtained in the HPC platform, although the execution times are higher. Figure 5a shows the FM performance on the Xavier’s CPU and GPU, running the first experiment. Its behavior is similar to the one obtained in the HPC platform. For executions with number of columns lower than 32, the CPU shows better performance as the workload is too light. Once this threshold is reached, the GPU outperforms the CPU by a margin of 2×. Figure 5b shows the FM performance, running the experiment 2. In this case, the GPU implementation offers better performance than the CPU as the number of rows is increased. For very small workloads, the differences in performance between CPU and GPU are very insignificant. However, when the maximum number of rows being studied is reached, an speed-up factor of 2× is achieved.

### 4.3. HPC vs. Edge Computing Platform

This section compares the HPC and edge computing platforms. Although both of them are heterogeneous systems (i.e., CPU + GPU), they are designed for different purposes. The HPC platform is power-hungry; thus, its CPU and GPU offer high performance ratios. However, the edge computing platform is designed for energy efficiency with a reduced power budget. With that in mind, Table 2, Table 3 and Table 4 show the performance of these architectures, running the three clustering algorithms targeted.

Table 2 shows the k-means performance evaluation on both targeted platforms and running the Experiment 1. The GPU code executed on the HPC platform obtains up to 4× speed-up factor compared to its edge computing counterpart version. Indeed, the GPU available on the HPC platform (i.e., NVIDIA GPU GeForce RTX 2080 Ti) is much more powerful than the GPU available on the edge computing device, which only has a stream multiprocessor with 512 CUDA cores. However, performance differences reach this level for heavier clustering, i.e., 100,000 rows dataset. For smaller workloads, the differences are significantly reduced. For instance, 100- and 1000-row datasets run even faster on the Xavier where the runtime overhead is lighter than in the HPC infrastructure. To sum up, HPC infrastructure requires higher computational workloads to hide its overall runtime overhead, but, once hidden, significant performance differences are obtained.

Table 3 shows performance figures of the FCM algorithm. In this case, the performance difference between platforms is higher. The sequential implementation of FCM algorithm on the HPC platform exceeds 35× of speed-up factor compared to the CPU in the edge computing platform. Regarding GPU versions, the performance differences are close to 25× speed-up factor in favor of the HPC platform. In general, the FCM algorithm gets higher performance when running on the HPC platform, since this algorithm is very expensive from a computational point of view.

Table 4 shows performance figures of the FM algorithm. The scalability of CPU and GPU implementations between both platforms is similar to k-means’ scalability. Again, smaller datasets (i.e., 100 rows) run even faster in the Xavier as they are very lightweight. However, the performance differences between HPC and edge computing platforms increase along with the number of rows, reaching up to 3× speed-up factor for the sequential code and 10× speed-up factor for the CUDA counterpart version. As in the case of the k-means algorithm, the GPU code obtains a greater benefit on the HPC platform for very heavy workloads. The computational differences between HPC and edge computing platforms in terms of GPUs are very noticeable, as mentioned above.

### 4.4. Energy Consumption Evaluation

**Figure 6:**
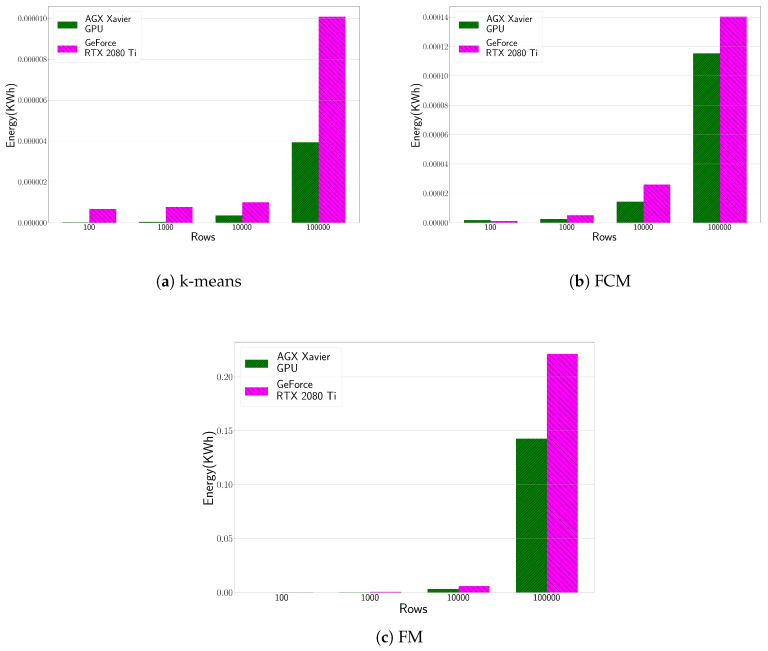
Energy consumption (in KWh) evaluation of the HPC and edge computing platform for the CUDA-based clustering implementations (i.e., k-means, FCM and FM). We focus on the GPU plugged on the HPC platform (NVIDIA GeForce RTX 2080Ti) and the whole system for AGX Xavier.

Figure 6 shows the energy consumption evaluation of the HPC and edge computing platformsw, running the three clustering algorithms targeted. The executions times are the same than those presented in Table 2, Table 3 and Table 4, respectively. Generally speaking, the NVIDIA Jetson Xavier is more energy efficient than the NVIDIA GeForce RTX 2080 Ti. Although the GeForce is faster than Xavier as shown previously, its power consumption is much higher, i.e., GeForce registers 270 W, while the Jetson registers between 8 W–10 W. The most striking result to emerge from the data is that these edge computing devices are a compelling alternative in terms of energy efficiency, even in scenarios where the computational cost is too high. Actually, energy savings when running the k-means algorithm in the edge computing platform reaches up to 150%, 16% for FCM where the performance gap is not so high and finally 80% for the FM algorithm. Moreover, the power consumption of the HPC infrastructure only measures the power consumed by the GPU, rather than the power consumption of the entire platform as is the case with NVIDIA Jetson Xavier. This can even increase the latter’s benefit in terms of energy consumption.

## 5. Conclusions and Future Work

The generation of a novel IoT application must be based on the efficient analysis of the data deluge generated. Clustering techniques are unsupervised learning methods that involve the grouping of data points and can be used to gain some valuable insights, such as extraction patterns and identify outliers, among others. However, these workloads are computationally expensive, limiting its use in real-world IoT applications. This article evaluates edge computing devices as a compelling alternative for running computationally expensive workloads, such as those within the umbrella of machine learning. Particularly, we focused on three widely used clustering algorithms techniques, such as k-means, FCM, and FM. We explored the use of CPUs and GPUs on HPC platforms (Intel + NVIDIA) and edge computing platforms (NVIDIA Jetson AGX Xavier). Our results show performance differences of up to 11× speed-up factor when the edge computing device uses its low-power GPU. In addition, the use of edge computing platform reports great energy savings which are in the range of 16% and 150%, depending on the computational differences between both architectures. In fact, these results confirm that the inclusion of GPU accelerators at the edge is a compelling alternative for bringing the AI challenge to autonomous IoT infrastructures.

The conjunction of edge computing and AI embraces novel IoT applications, which are still at a relatively early stage. We recognize that we have only tested a relatively simple variant of this solution that is designed for a particular combination of hardware and algorithms. But, with the advent of 5G technology, we definitely think that designing low-power solutions can reduce the overall’s power consumption by maintaining the performance gains for edge devices. Moreover, there are many other types of data science algorithms still to explore, and it is a potentially fruitful area of research. We hope that this paper stimulates further discussion and work.

## Figures and Tables

**Table 1:** Main features of the targeted clustering algorithms.

Algorithm	Soft Clustering	Nº of Cluster Pre-Fixed	Requisites
K-Means	No	Yes	CWS Clusters
FCM	Yes	Yes	CWS Clusters
FM	Yes	n.a.	none

**Table 2:** Comparison of the execution time (in seconds) of the GPU and CPU implementations of the k-means algorithm between the HPC and edge computing platforms.

Rows	AGX Xavier		HPC Platform		Speed-Up Factor	
					(HPC vs. Edge)	
	CPU	GPU	CPU	GPU	CPU	GPU
100	0.004	0.007	0.065	0.035	0.1	0.2
1000	0.112	0.020	0.104	0.040	1.1	0.5
10,000	1.335	0.159	0.587	0.052	2.3	3.1
100,000	19.944	1.761	7.544	0.469	2.6	3.8

**Table 3:** Comparison of the execution time (in seconds) of the GPU and CPU implementations of the FCM algorithm between the HPC and edge computing platforms.

Rows	AGX Xavier		HPC Platform		Speed-Up	
	CPU	GPU	CPU	GPU	CPU	GPU
100	107.792	0.510	2.988	0.089	36.1	5.8
1000	28.262	2.246	1.033	0.093	27.4	24.2
10,000	44.277	11.584	1.414	0.479	31.3	24.2
100,000	329.851	71.835	8.424	2.876	39.2	25.0

**Table 4:** Comparison of the execution time (in seconds) of the GPU and CPU implementations of the FM algorithm between the HPC and edge computing platforms.

Rows	AGX Xavier		HPC Platform		Speed-Up	
	CPU	GPU	CPU	GPU	CPU	GPU
100	0.045	2.663	0.126	3.015	0.4	0.9
1000	5.512	116.918	0.976	20.840	5.6	5.6
10,000	735.811	2379.008	218.281	214.364	3.4	11.1
100,000	118,556.281	83,134.25	48,699.251	7968.036	2.4	10.4
